# A new model of implant-related osteomyelitis in the metaphysis of rat tibiae

**DOI:** 10.1186/s12891-016-1005-z

**Published:** 2016-04-08

**Authors:** Norbert Harrasser, Johannes Gorkotte, Andreas Obermeier, Susanne Feihl, Melanie Straub, Julia Slotta-Huspenina, Ruediger von Eisenhart-Rothe, Walter Moser, Philipp Gruner, Michael de Wild, Hans Gollwitzer, Rainer Burgkart

**Affiliations:** Clinic of Orthopedics and Sports Orthopedics, Klinikum rechts der Isar, Technical University of Munich, Ismaninger Str. 22, 81675 Munich, Germany; Institute for Medical Microbiology and Immunology, Technical University of Munich, Trogerstr. 30, 81675 Munich, Germany; ATOS Clinic, Effnerstr.38, 81925 Munich, Germany; Institute of Pathology, Klinikum rechts der Isar, Technical University of Munich, Ismaninger Str. 22, 81675 Munich, Germany; Atesos medical AG, Schachenallee 29, CH-5000 Aarau, Switzerland; Medicoat AG, Almuesenacherstrasse 2a, CH-5506 Maegenwil, Switzerland; University of Applied Sciences and Arts Northwestern Switzerland (FHNW), School of Life Sciences, CH-4132 Muttenz, Switzerland

**Keywords:** Implant-associated infections, Rat, Screw, Metaphysis, *Staphylococcus aureus*

## Abstract

**Background:**

Animal models serve as an important tool to understand peri-implant infection. Most of the models use high bacterial loads (>10^4^ colony forming units, CFU) to provide high infection rates. Therefore these animals evolve rather similarly, making comparison between groups and statistical analysis possible. On the other hand, to mimic clinical constellation of surgery-related infections the use of low amounts of bacteria would be more advantageous.

**Methods:**

We developed a metaphyseal rat model of peri-implant bone infection with low amount of bacterial loads (10^2^ and 10^3^ CFU of *Staphylococcus aureus*) and investigated osseointegration of the implants coated with hydroxyapatite (HA) and low-dosed HA-silver (HA-Ag). Non-infected implants served as controls. After 6 weeks rats were sacrificed and implants evaluated for osseointegration and infection.

**Results:**

Infection of implanted devices was reliably induced, independently whether 10^2^ or 10^3^ CFU of *S. aureus* were inoculated and HA or HA-Ag coated implants were used. No systemic infection was present in any of the animals at the time of sacrifice, and no animal developed acute infection requiring premature sacrifice. All CFU counts of the implant and the bone at sacrifice were significantly higher than the inoculated load (*p* < .05). All sterilely inserted implants showed excellent osseointegration and no infection.

**Conclusions:**

Our present study of a rat tibia model reliably induced osteomyelitis in the metaphysis with low-doses of bacteria. The addition of low-dosed Ag to the implant coating was not able to reduce the infection rates. The results demonstrate that it is possible to develop a model of implant-related osteomyelitis in rats with low amounts of bacteria to better mimic clinical constellations. No other promoters of infection besides insertion of the screw implant were used in this model.

## Background

Peri-implant infections (PII) are serious complications in orthopedic surgery, representing a significant healthcare and economic burden [1]. Management of these infections often requires multiple staged surgeries and the use of antibiotics as a supportive therapy for eradication [2, 3]. Up to day several factors in the pathogenesis and treatment of implant-related bone infections are still unclear. Animal models are therefore considered helpful for understanding mechanisms of implant-associated osteomyelitis as well as in-vivo testing of potential anti-infective implant coatings and antibiotics. Consequently, a number of different models of PII have been developed [4–9]. A major problem in this context is that several crucial parameters (e.g. bacterial load, bacterial strains, implant configuration, implant location) differ between the various models making comparison among each other difficult. The amount of seeding bacteria is subject to controversial debates. To promote signs of infection, usually bacterial counts far beyond 10^3^ colony forming units (CFU) have been used [4, 8, 10, 11]. This creates high rates of infection in nearly all animals but on the other hand is not very helpful to mimic clinical constellation of surgery-related infections. Another disadvantage of various animal models is the fact that osseointegration usually cannot be evaluated. If K-wires, plates or even artificial joints are used as implants removal of these devices usually can’t be quantified and no statement can be made whether osseointegration took place or not. Finally, many documented animal models of peri-implant bone infections used diaphyseal implant positioning. However, metaphyseal fixation is more common in the clinical use of orthopedic implants [6, 10, 12, 13].

Hence, the aim of the present study was to evaluate a novel animal model mimicking infection with low amounts of bacterial inocula at the level of the tibial metaphysis. For generation of implant-associated infection the amount of bacteria was tested as an independent parameter *(Staphylococcus aureus;* 10^2^ vs. 10^3^ CFU). Additionally, investigation of osseointegration of the device was carried out in a semiquantitative manner.

## Methods

### Implants

**Fig. 1:**
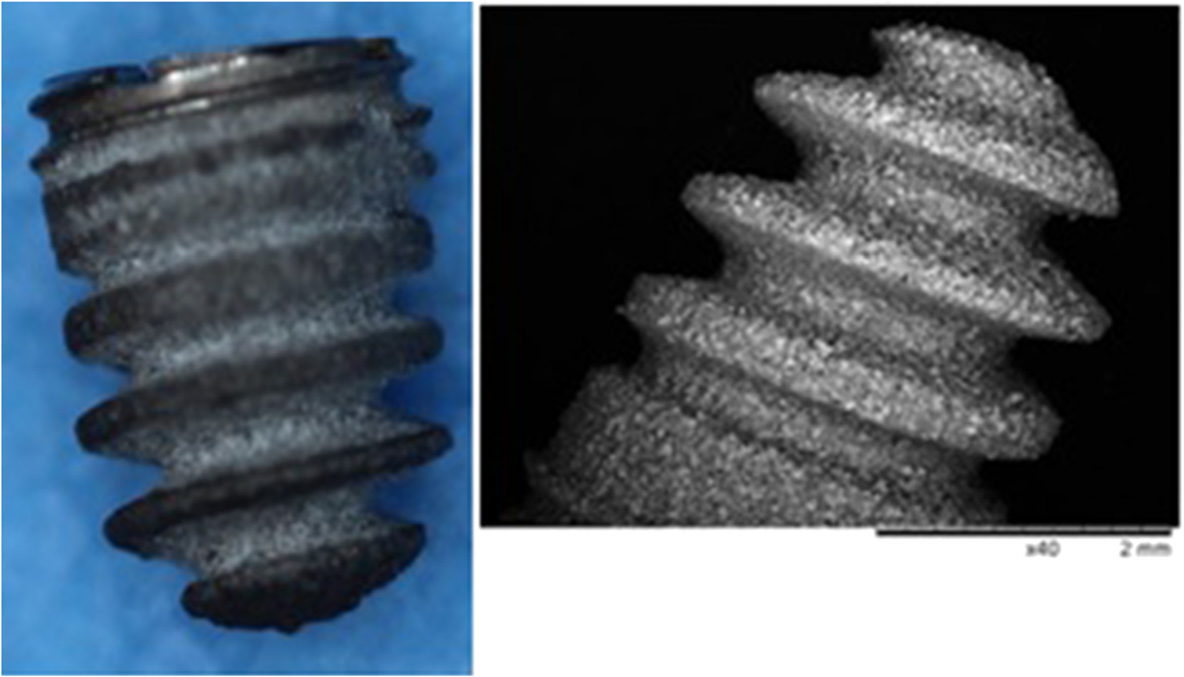
SEM image of the coated screw implant (diameter = 3.5 mm; length = 5 mm)

**Fig. 2:**
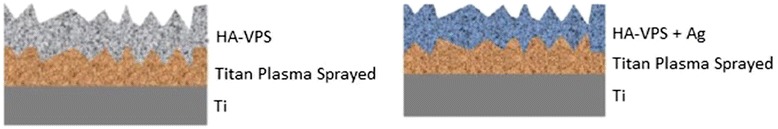
Coating structure of HA (*left*) and HA-Ag (*right*)

**Fig. 3:**
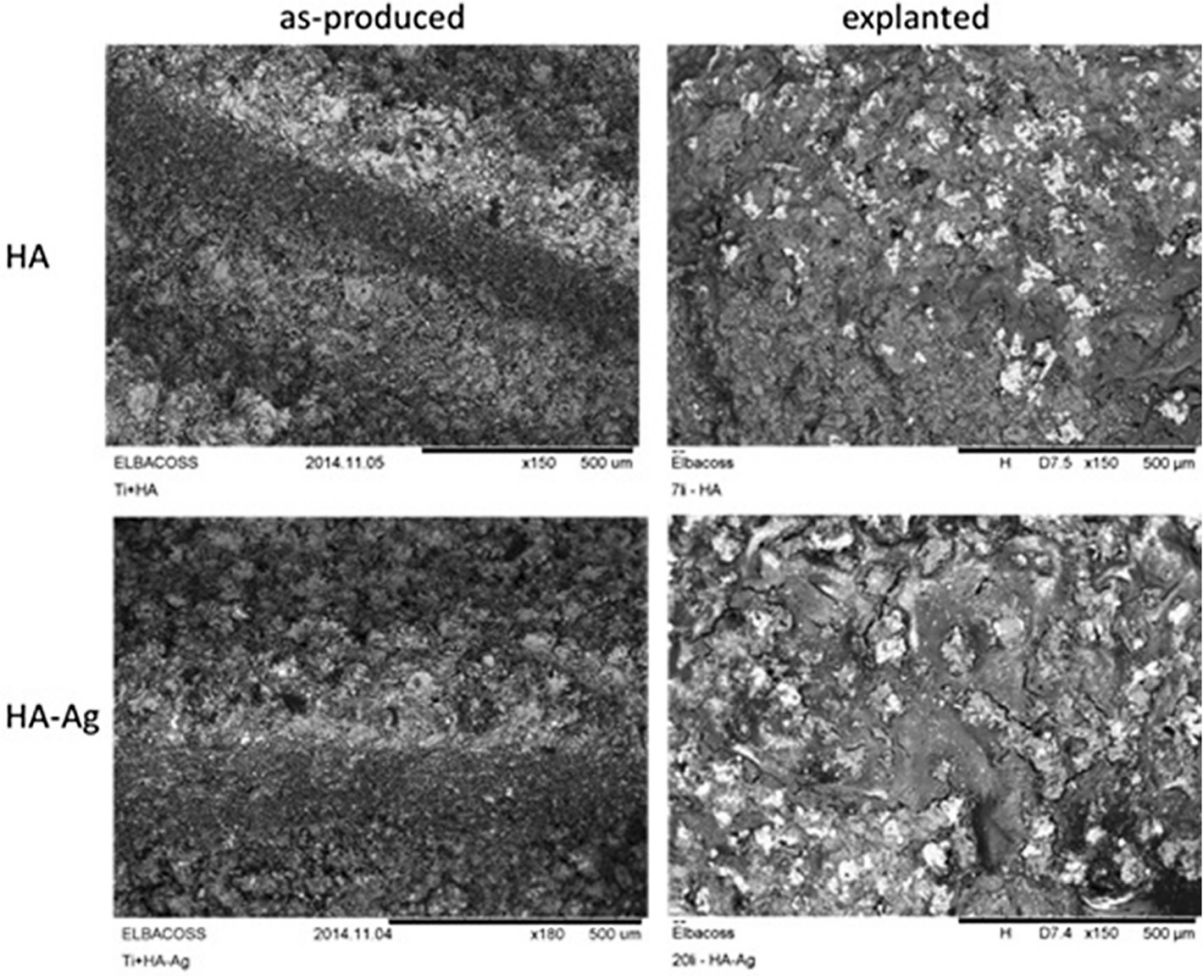
SEM analysis (backscattered electron detector) of both surface types before and after implantation

The implant used in the present study was a custom-made titanium (Ti6Al4V) screw (Fig. 1). The screw was coated with HA and HA-Ag (Atesos medical AG, Aarau, Switzerland; Medicoat AG, Mähenwil, Switzerland) according to a manufacturing process previously described (Fig. 2) [14]. Briefly summarized, a modified technique of Vacuum Plasma Spraying (VPS) coating was used so that a thickness of ~100 μm was achieved for the coatings. The HA-Ag coating contained low amounts of Ag (45 ppb). The morphology of the surface was analyzed by Scanning electron microscope (Hitachi TM-3030Plus) using secondary electron detector. The surface roughness was determined on the apical position of the implant by Confocal Laser Scanning Microscope (Lext OLS 3100, Olympus) equipped with a 50× objective. The R_a_-1D-values were determined by averaging *n* = 10 profiles of length 260 μm, using a cut-off wave-length of 26 μm. The SR_a_ and SR_z_-2D-values were defined of *n* = 5 areas of 295 × 20 μm^2^ also applying a cut-off wave-length of 26 μm. Due to the HA-VPS-coating, the surface of both implants showed a rough surface morphology with consistent arithmetic average roughness R_a_ of 2.49 ± 0.62 μm for group I (HA) and 2.21 ± 0.47 μm for group II (HA-Ag) surface moodification. The 2D surface rouhness parameters SR_a_ (arithmetic average roughness) and SR_z_ (maximum height) listed in Table 1 were also identical for both groups. The coarse surface topography was observed in the SEM image showing a homogeneous VPS-coating for either groups (Fig. 3).

**Table 1:** Surface roughness (mean score values ± standard deviation) of the implant

	Ra [μm]	SRa [μm]	SRz [μm]
Group I (HA)	2.49 ± 0.62	2.98 ± 0.24	117.38 ± 10.74
Group II (HA-Ag)	2.21 ± 0.47	3.22 ± 0.80	105.03 ± 12.07

### Bacterial strains and preparation of inocula

The bacterial strain selected in the study was one of the most common causative pathogens associated with PII, namely *S. aureus (*ATCC25923, LGC Standards GmbH, Wesel, Germany*).* The strain was routinely cultured in Columbia Agar with 5 % sheep blood (Becton Dickinson, Heidelberg, Germany) at 37 °C overnight before testing. Bacteria were then harvested by centrifugation, rinsed, suspended, diluted in sterile phosphate buffered saline (PBS) and adjusted by densitometry (MacFarland Densimat™, BioMérieux, Marcy l’Etoile, France). For the study every suspension with its known bacterial concentration was diluted with NaCl to reach the targeted value for bacterial concentration (Group I-IIA: 10^2^ CFU/10 μl, Group I-IIB: 10^3^ CFU/10 μl). In order to obtain log-phase growth over-night freezing of strains was strictly avoided and incubation of bacteria at 37 °C throughout the preparation time was conducted. To quantify the bacterial load prior to inoculation serial dilutions of the residual suspensions were incubated on agar plates for 48 h at 37 °C, and the number of inoculated viable cells was then calculated.

### Animals and surgical procedure

The study was approved by the Animal Experimentation Ethics Committee of Bavaria (Reg. No. 146-14) and was conducted with reference to the OECD Principles of Good Laboratory Practice. According to the committee’s recommendation that the number of experimental animals be minimized, the sample was limited to 12 rats per group and both tibiae were used for study purposes. Hence, 24 male, 5-month-old Wistar rats (Charles River Laboratories, Sulzfeld, Germany) with a mean body weight (BW) of 378.6 g (range: 353–401 g) were used. For acclimatization, the animals were delivered to the animal facility at least 1 week prior to treatment. Animals were housed in cages (2-5 animals) at normal room temperature and daylight illumination with ad libitum access to food and water.

Surgery was performed under general anesthesia induced by weight-adapted intramuscular injection of Medetomidin (150 μg/kg BW), Midazolam (200 μg/kg BW) and Fentanyl (5 μg/kg BW). Blood was collected by puncture of the tail vein prior to surgery.

**Fig. 4:**
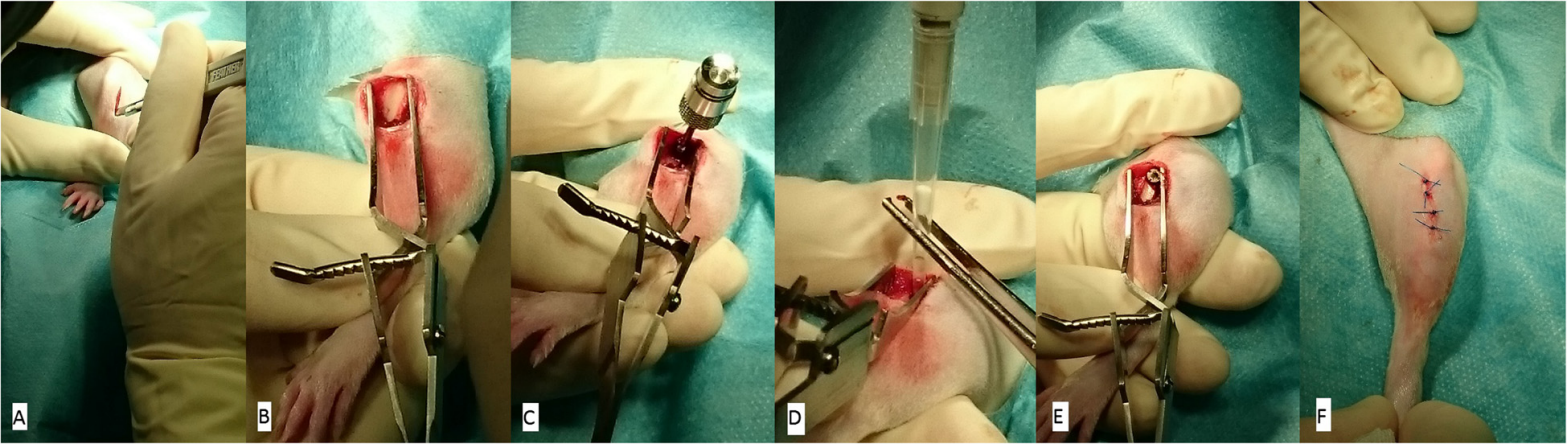
Operative procedure (left tibia): **a** Skin incision at the anterolateral aspect of the proximal tibia, **b** visible proximal tibia after blunt dissection of soft tissues, **c** drilling and tapping of unicortical hole, **d** injection of PBS (left tibia = control site), **e** insertion of implant (screw not yet full countersinked), **f** skin closure with intracutaneous and interrupted sutures

Animals were prepared for surgery as follows (Fig. 4): Both hind legs were shaved, antisepticized with povidone–iodine, and dried. Bodies were covered with sterile sheets except for the hind legs. A skin incision (length, 1–2 cm) was made over the proximal lateral tibial metaphysis. A unicortical hole with a depth of ~8 mm was drilled using a 1.8 mm diameter drill bit. The drill hole was then tapped with a custom-made stainless steel tap and dried with gauze. After removal of the gauze 10 μl of either sterile PBS (left tibia = control) or PBS/*S. aureus* in the entitled concentrations (right tibia = infected) were injected into the hole with a 25 μl microsyringe (Hamilton, Reno, NV). The implant was inserted into the cavity immediately after injection of the solutions using a dedicated instrument. Soft tissue was irrigated with saline solution, the fascia was closed using absorbable suture material (Vicryl rapid, Ethicon Inc., Cincinnati, USA; size 6-0), the skin was closed by continuous intracutaneous (4-0 Monocryl, Fa. Ethicon, Norderstedt, Germany) and interrupted sutures (4-0 Prolene, Fa. Ethicon, Norderstedt, Germany). Anesthesia was antagonized after surgery by a subcutaneous injection of 750 μg/kg BW atipamezol (Alzane, Pfizer, Berlin, Germany), 200 μg/kg BW flumazenil (Flumazenil-ratiopharm, Ratiopharm, Ulm, Germany) and 120 μg/kg BW naloxone (Naloxon-Ratiopharm, Ratiopharm, Ulm, Germany). Metamizole (110 mg/kg BW) was administered subcutaneously (s.c.) at the beginning of the surgery. Additionally, buprenorphine (0.05 mg/kg BW) was administered at the end of the surgical procedure immediately before the wound closure was completed. Postoperative pain control was also carried out with buprenorphine (0.05 mg/kg BW s.c., 2 x daily) for 2 days. In addition, meloxicam was given s.c. immediately postoperatively and then followed for days 1–3 every 24 h at a dose of 1 mg/kg BW.

Rats were divided into two groups according to the type of implant inserted (Table 2).

**Table 2:** Main features of testing groups

Group	IA	IB	IIA	IIB
Implant coating	HA		HA-Ag	
Right tibia: *S. aureus* (CFU)	10^2^	10^3^	10^2^	10^3^
Left tibia: *S. aureus* (CFU)	0	0	0	0
Number of animals	6	6	6	6

### Sacrifice

The animals were sacrificed after 42 days. Under general anesthesia 3 ml of blood was collected by puncture of the right atrium and a lethal dose of pentobarbital (80 mg/kg BW) was injected. Tibiae of both hind legs, starting with the control site (left) were dissected under sterile conditions and further investigations were conducted.

### Radiographic evaluation

Radiographs were taken in two planes immediately after implantation and on the day of sacrifice. For X-rays, digital films (DLR Cassette, Digiscan 2H/2C, Siemens) and a Mobilett Plus X-ray unit (Siemens AG, Erlangen, Germany) were used. Three regions of interest (ROIs) were determined (R1, epiphysis; R2, metaphysis with implant; R3, proximal diaphysis) and investigated separately for [5]:

1. Periosteal reaction
2. Osteolysis
3. Soft-tissue swelling
4. Sequestrum formation
5. Implant loosening

Parameters 1–4 ranged from 0 (absent), 1 (mild) to 2 (severe), and were evaluated separately for ROIs 1–3. Parameter 5 was evaluated for present or not present. If the implant was loose the average score for parameters 1–4 was doubled. The maximum score possible was 48 (3 ROIs × 4 parameters × 2 points × 2 for loose implant). We did not perform radiological evaluations in the acute phase (first weeks after operation) as most radiological signs of bone infection are not apparent in this phase and therefore an additional general anesthesia for the animals would be of minor scientific benefit [6].

### Evaluation of osseointegration

**Fig. 5:**
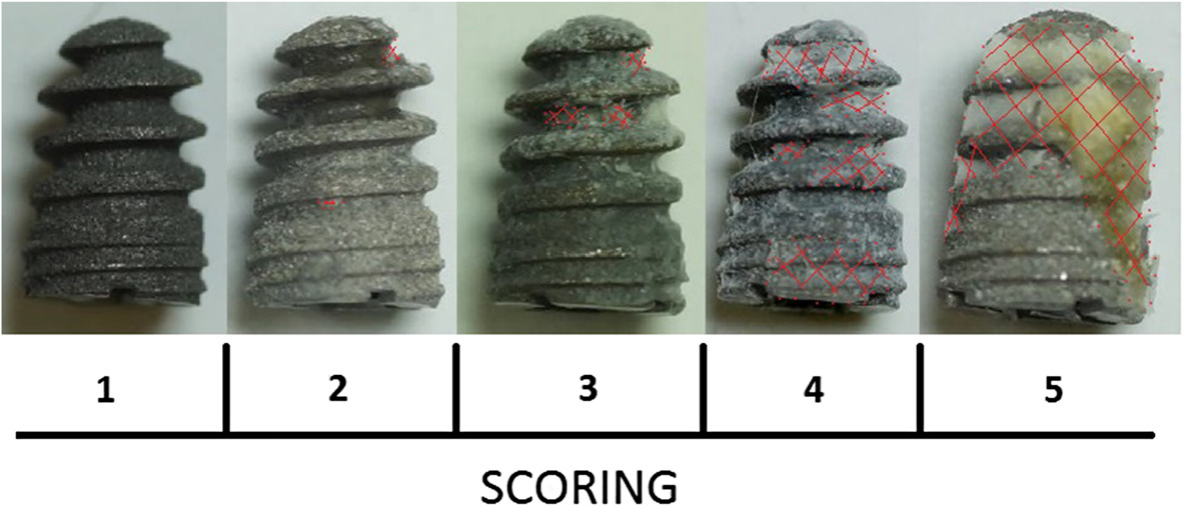
Scoring of osseointegration (Note: shaded area in red indicates bone on implant surface after explantation of the screw)

The screw was removed manually using a sterile custom-made screwdriver and afterwards investigated macroscopically for attached bone remnants. Resistance-to-removal (RTR) was evaluated semiquantitatively, attached bone remnants (ABR) were scored quantitatively. The following scoring system ranging from 1–5 points for either items (RTR and ABR) was applied (Table 3, Fig. 5). Means of either values (RTR and ABR) were summarized and osseointegration-score was calculated accordingly (range: 2–10). Additionally, two sterilely inserted implants of group I (HA) and II (HA-Ag) were randomly chosen and not removed but histologically evaluated for osseointegration [15]. Specimens were sectioned by a diamond band saw (Exakt 300P) in the middle of the implant, glued to a support, and sectioned again, so that a 200 mm-thick sample from the middle was attached to the support. The thickness of this sample was further reduced to 40–60 mm by a grinding machine (Exakt 420 CS). To visualize tissues, the samples were surface stained by toluidine blue.

**Table 3:** Scoring system for resistance-to-removal (RTR) and attached bone remnants (ABR)

Score	RTR	ABR
1	Loose screw can be removed with forceps only	No bone detectable on implant
2	Little resistance (only tip of 2 fingers needed to hold screwdriver)	Traces of bone remnants on implant
3	Moderate resistance (screwdriver held in pinch grip)	Thin bone layer on implant (10–25 % of surface coated with bone)
4	Strong resistance (like 3 but more power needed)	Moderate bone layer on implant (25–50 % of surface coated with bone)
5	Bone fracture with screw partly covered by bone	Thick bone layer on implant (>50 % of surface coated with bone)

### Microbiological evaluation

#### Examination of bacteriology swabs

Bacteriology swabs from the subcutaneous tissue and the implant site were obtained bilaterally of all animals. Swabs were moistened with one drop (20 μl) of sterile PBS, then evenly streaked onto a plate each of Columbia-Agar and Schaedler-Agar, and immersed in thioglycollate broth. The solid and liquid media were incubated for 48 h at the below mentioned conditions:

- Columbia-Blood-Agar (BD): 37 °C
- Schaedler-Agar (BD): 37 °C under anaerobic conditions
- Thioglycollate broth (prepared in-house): 37 °C

After 48 h the solid and liquid media were analyzed by conventional bacteriological techniques. The identity of *S. aureus* isolates was determined by catalase and coagulase testing or by matrix-associated laser desorption/ionization-time of flight mass spectrometer (MALDI-TOF, Bruker Corporation, Billerica, U.S) if the catalase/coagulase testing was unclear.

#### Examination of the implant

Implant-adhering bacteria were detached from the implant immersed in 1 ml PBS using low frequency ultrasound treatment (Sonorex digital 10P, Bandelin, Berlin, Germany: 5 min. at 80 % intensity). After the treatment, tubes were centrifuged at 4000 rpm for 10 min. at 4 °C temperature (Heraeus Varifuge 3.OR; Kendro Laboratory Products, Osterode, Germany). The supernatant was resuspended in 300 μl PBS. A serial tenfold dilution of this irrigated PBS was incubated on agar plates at 37 °C for 48 h, and the number of inoculated viable cells was then calculated.

#### Examination of peri-implant bone

Adhering soft tissues were removed from the tibia, and the remaining bone was ultrasonically irrigated in 10 ml of PBS for 5 min. A serial tenfold dilution of this irrigated PBS was incubated on agar plates at 37 °C for 48 h, and the number of inoculated viable cells was then calculated.

### Histological evaluation of infection

One randomly chosen tibia from each group (IA/B, IIA/B; one from the control site, one from the infected site = totally eight specimens) was fixed for two days in 5 % formaldehyde and decalcified in 5 % nitric acid solution. The tibia was embedded in paraffin and cut into 4 mm thick sections. Slices were stained with haematoxylin/eosin. Three ROIs according to the radiographical evaluation were analyzed on [7, 12]:

1. Infiltration of granulocytes
2. Sequestrum formation
3. Infiltration of mononuclear cells and bone marrow fibrosis
4. Enlargement of cortical bone
5. Erosion/destruction of cortical bone
6. General impression

Parameters 1 to 5 were scored with 0 (absent) or 1 (present). Parameter 6 was scored from 0 (absent), 1 (mild) to 2 (severe). The maximum score was 21. (3 × ROIs (parameter 1–5) × max. 1 point + 3 × ROI (parameter 6) × max. 2 points).

### Statistics

All results are presented as means ± standard deviation (SD). Statistical significance was computed using non-parametric methods and the method of closed testing procedure (Kruskal-Wallis test, Mann-Whitney U test, Wilcoxon test). *P* < 0.05 was considered statistically significant. Statistical tests were performed with use of SPSS (version 20.0; Chicago, Illinois).

## Results

### Clinical evaluation of animals

The animals recovered quickly after surgery and showed no signs of discomfort. There was no statistically significant difference in mean weight gain between the groups if the inoculated bacterial load was considered (Group I/IIA: 30 ± 11 g, Group I/IIB: 23 ± 17 g).

### Blood analyses

The course of hemoglobin (day of surgery vs. day of sacrifice) showed no significant difference between the groups throughout the experimental period. Further, infect parameters (white blood cell count, platelets) showed no significant elevation at day of sacrifice (Table 4).

**Table 4:** Number (mean ± standard deviation) of leucocytes = white blood cells (WBC), erythrocytes = red blood cells (RBC), and platelets in blood samples on day of surgery (day 1) and at sacrifice (day 42)

Parameter	Group IA (10^2^ CFU)		Group IB (10^3^ CFU)		Group IIA (10^2^ CFU)		Group IIB (10^3^ CFU)	
	Day 1	Day 42	Day 1	Day 42	Day 1	Day 42	Day 1	Day 42
WBC (×10^3^/l)	9,23 ± 1,98	7,53 ± 1,97	9,33 ± 3,71	6,05 ± 1,80	9,07 ± 1,98	7,87 ± 3,22	9,62 ± 2,62	8,57 ± 3,59
RBC (×10^6^/l)	8,27 ± 0,76	8,64 ± 0,95	7,13 ± 2,40	8,55 ± 1,08	8,21 ± 1,41	8,38 ± 0,67	8,19 ± 1,29	6,34 ± 5,66
Platelets (×10^3^/l)	834,5 ± 189,25	628,33 ± 237,56	717,83 ± 406,97	599,83 ± 528,47	912,17 ± 135,77	649,00 ± 321,04	763,33 ± 290,16	751,50 ± 343,20

Note: No statistical significant difference regarding various parameters of different groups was found between day 1 and 42

*p < 0.05 in groups between day 1 and day 42

### Microbiological evaluation

#### Day of surgery

Plate counts of bacterial suspension revealed an average number of inoculated viable cells for the right tibiae of 1.43 × 10^2^ CFU/10 μl (±16.1) for Group A (Target: 10^2^ CFU), and 0.89 × 10^3^ CFU/ml (±40.5) for Group B (Target: 10^3^ CFU). The difference between the two groups was statistical significant.

#### Day of sacrifice

50 % of cultures obtained from bacteriological swabs from the implant surface, and ~20 % of the swabs taken from the subcutaneous tissue in groups of infected animals were positive for *S. aureus* (Table 5). On the other hand, 100 % of cultures from bone and implant were positive. Coagulase/catalase testing and MALDI-TOF evaluation revealed the same strain as inoculated in all tested animals. Average bacterial counts from the bone site of infected groups (I: HA, II: HA-Ag) were significantly lower compared to bacterial counts from the implant site (Table 6). Regarding the bacterial amount harvested from the osseous site highest counts were found in tibiae from group IIB, followed by group IIA, group IA and group IB. Taken into account that groups with HA-Ag (IIA/B) did not show less bacterial growth at sacrifice compared to groups with HA (IA/B) a correlation between inoculation loads of groups A (10^2^ CFU) and groups B (10^3^ CFU) and loads at sacrifice was not found. Therefore, the amount of inoculated staphylococci on day of surgery did not correlate with the amount of bacteria at day of sacrifice (Table 6).

**Table 5:** Microbiological results and bone weight of right tibiae (infected) determined on day of sacrifice; ^a^ positive tested on *S. aureus*

		Groups			
		IA (HA, *n* = 6**)**	IB (HA, *n* = 6)	IIA (HA-Ag, *n* = 6)	IIB (HA-Ag, *n* = 6)
Cultures of subcutaneous smears	Positive^a^	1	1	2	1
Cultures of implant surface smears	Positive^a^	2	3	3	4
Cultures of implant	Positive^a^	6	6	6	6
Cultures of peri-implant bone	Positive^a^	6	6	6	6
Bone weight [mg]		504 ± 27	498 ± 37	456 ± 67	546 ± 78

**Table 6:** CFU values (mean ± standard deviation) at implantation day (Inoculum) and day of sacrifice of the implant and the periprosthetic bone; *p*-values are given for comparison Inoculum/Implant and Implant/Bone

Groups	Target	CFU/10 μl				
		Inoculum	Implant	*p*-value (Inoculum vs. Implant)	Bone	*p*-value (Implant vs. Bone)
IA (HA)	10^2^	1.16x102 ± 75	116.1x 103 ± 112x10^3^	<.05	21.5x10^3^ ± 29.3x10^3^	<.05
IIA (HA-Ag)	10^2^	1.71x102 ± 52	392.2x10^3^ ± 641x10^3^	<.05	30.2x10^3^ ± 38.3x10^3^	<.05
IB (HA)	10^3^	0.97x103 ± 221	302.2x 103 ± 298x10^3^	<.05	8.2x10^3^ ± 3.9x10^3^	<.05
IIB (HA-Ag)	10^3^	0.83x103 ± 278	119.3x 103 ± 161x10^3^	<.05	60.9x10^3^ ± 55.1x10^3^	<.05

In control groups I-IIA/B for the left tibiae no bacterial growth could be observed on any implant cultures or from sonicated fluids of the bone. Additionally, all smears were found to be negative.

### Histological evaluation of infection

**Fig. 6:**
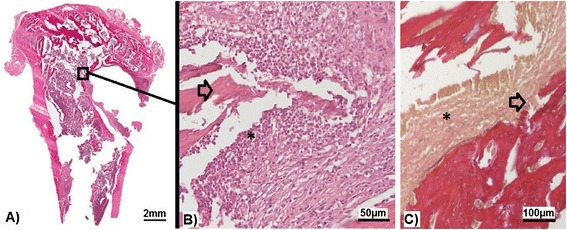
**a** Overview over epi-metaphysis and proximal diaphysis of a right tibia (inoculation with 10^2^ CFU), showing subacute osteomyelitis (H&E stain). **b** 25 x magnification of picture A, showing inflammatory infiltrate (*asterisk*), containing mononuclear cells and granulocytes, and bone necrosis (*black arrow*) (H&E stain). **c** Bone erosion (*black arrow*) due to inflammatory infiltrate (*asterisk*) (15 x magnification, van Gieson’s stain)

All retrieved specimens of the control sites of groups I-IIA/B (inoculated PBS) showed no signs of bone infection (Table 7). In these groups large amount of newly formed bone around the implants and in contact with the implant surface were observed suggesting good biocompatibility of the implants. All histological slices from infected sites showed typical signs of chronic bone infection (Fig. 6). Infection signs were only detectable in ROIs 1 (epiphysis) and 2 (metaphysis). Mean score value of control sites (PBS) was zero. Due to the small sample size per group no statistical analysis was conducted (Table 7).

**Table 7:** Outcome of radiographic and histologic assessment and osseointegration (mean score values ± standard deviation)

Groups	IA (HA)		IB (HA)		IIA (HA-Ag)		IIB (HA-Ag)	
	Left	Right	Left	Right	Left	Right	Left	Right
CFU	0	10^2^	0	10^3^	0	10^2^	0	10^3^
Radiographic score	0 ± 0	16.5 ± 6.4*	0 ± 0	15 ± 5*	0 ± 0	15.8 ± 4.8*	0 ± 0	16.5 ± 3.3*
Histologic score	0	16	0	13	0	6	0	9
Osseointegration score	8.7 ± 0.5	5 ± 0.5*	9.7 ± 0.4	4.7 ± 0.5*	9.3 ± 0.5	5.3 ± 0.5*	9 ± 0.5	4.3 ± 0.4*

*p < 0.05 between right (infected) and left (sterile) tibiae of each group

### X-ray examinations at day of sacrifice

**Fig. 7:**
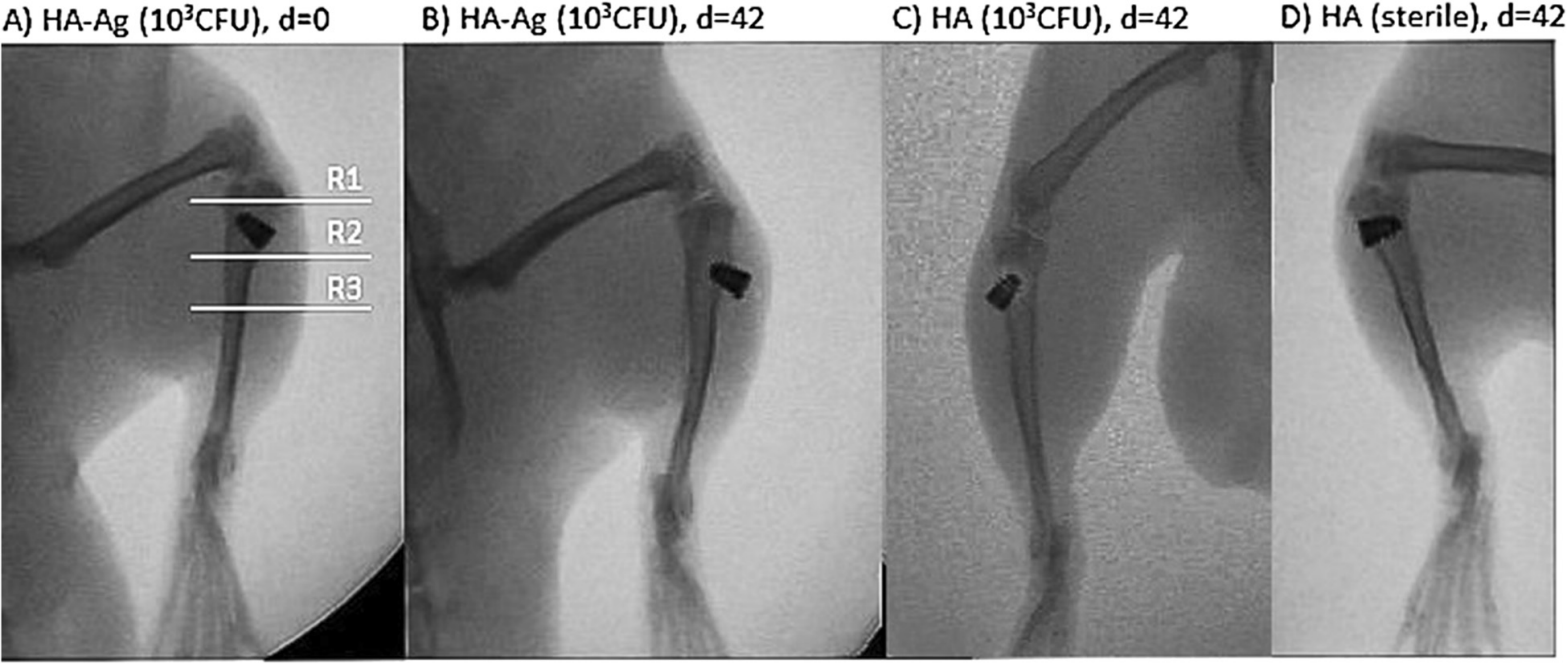
X-rays of tibiae in a.-p.-view at day of implantation (d = 0) and at day of sacrifice 6 weeks postop (d = 42). White lines mark regions of interest separately assessed for scoring. R1, epiphysis; R2, metaphysis; R3, proximal diaphysis. **a** and **b** Right tibia of infected animal showing signs of infection with osteolysis, and loose implant at day of sacrifice. **c** and **d** Left tibia (sterile) of an animal with no radiographic signs of osteomyelitis or implant loosening

X-rays of all infected sites revealed radiographic signs of osseous destruction in ROIs 1 (epiphysis) and 2 (metaphysis). No signs of infection were detected in ROI 3 (proximal diaphysis). Osteolysis, soft-tissue swelling and implant loosening were the most common findings, sequestrum formation and periosteal reaction on the other hand was never observed. In contrast to infected sites, X-rays of the control sites showed no signs of osteomyelitis (Fig. 7, Table 7).

### Osseointegration

**Fig. 8:**
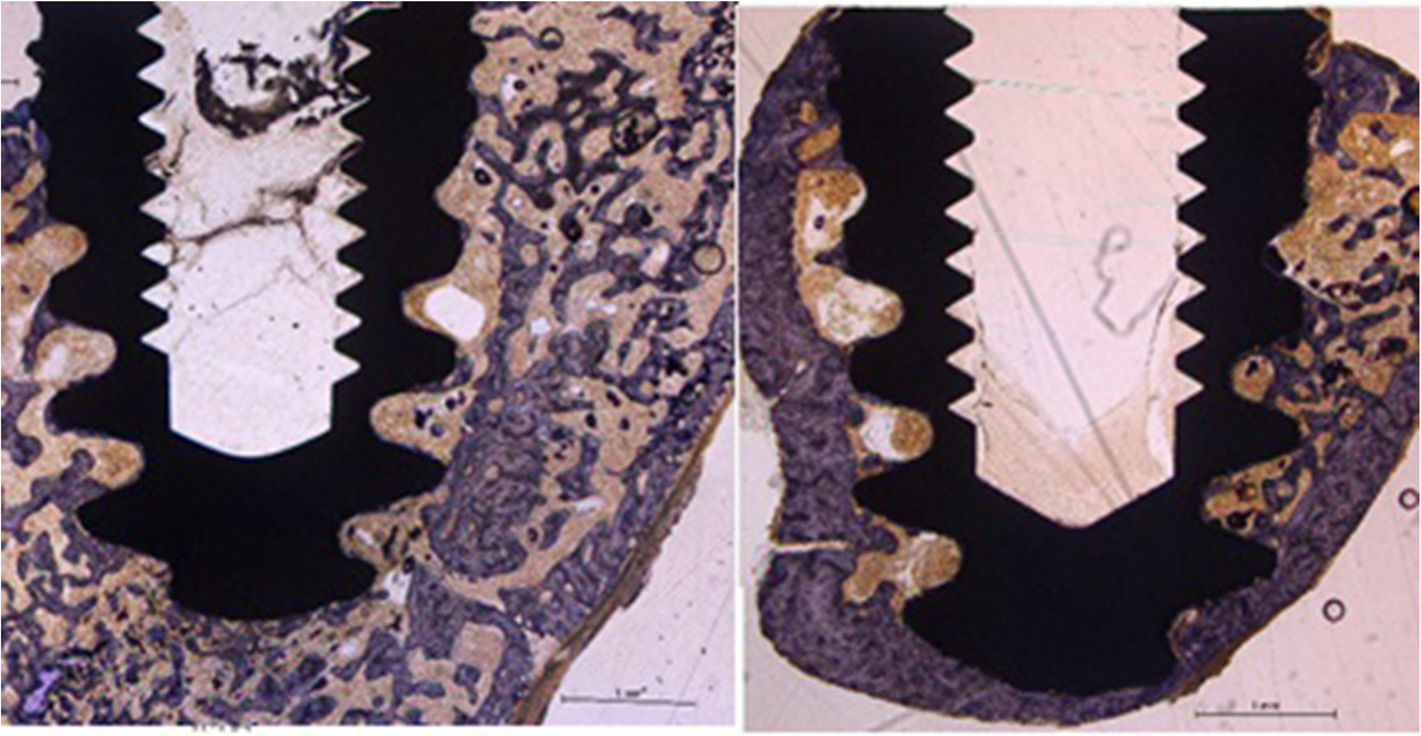
Osseointegration examined by histology. The surface of the implants is entirely coated by a thin layer of newly formed bone (*blue*) which is reached by trabeculae from the cancellous bone; HA (*left*), HA-Ag (*right*)

All implants of the control site showed excellent osseointegration with average score values ranging from 4 to 5. All infected sides on the other hand showed poor resistance to screw-out. Values ranged from 2 to 3. The differences between the groups were statistically significant (Table 7). Evaluation of osseointegration by histology showed broad bone integration throughout the whole surface of HA and HA-Ag implants (Fig. 8).

## Discussion

Many models of implant-associated infection in rats, mice, and rabbits have some significant limitations: Firstly, several studies investigated intramedullary nail implants or plates positioned at the level of the diaphysis and therefore restricted their findings mainly on situations from trauma surgery. However, a majority of bone and joint implants are fixed in the metaphyseal region [11, 16–18]. Secondly, bacterial loads used in several models are usually high and therefore don’t mimic clinical constellations [5, 7, 12, 16]. Thirdly, few infection models exist where osseointegration of implants can easily be investigated [9]. Therefore, the aim of the present work was to create an animal model where these limitations are overcome and findings are conferrable to infections associated with orthopedic implants. Additionally, impact on osseointegration and antibacterial effect of a low concentrated antibacterial coating on the implant was investigated.

The design of the implant we chose in the present study has some important features (Fig. 1): The diameter of the most superficial threads is larger than the diameter of the drill and the thread cutter, and therefore allows the implant to be implanted “press-fit”, the most common used method of fixing cementless orthopedic devices. In addition, the cone-shaped profile provides sealing of the drilled hole with the inserted bacteria. In several models bone wax, fibrin glue or dental gypsum are applied to seal the site of application in order to prevent bacterial leakage into the surrounding tissue [4, 19, 20]. This leakage is a major problem if K-wires or other rods are inserted into the medullary cavity of long bones, since bleeding out of the bone can lead to a washing-out of bacteria that have been inoculated previously. Additionally, the implant we used is solid at the top and not hollow as described by other authors [4]. The disadvantage of hollow implants, besides the fact that they provide a contaminated “dead space” not accessible for host defense mechanisms, is that again bacteria can be washed-out by bleeding from the bone site. To avoid this, again the hole has to be sealed, e.g. by bone wax, which on the other hand is another foreign material potentially influencing bacterial growth. Finally, the placement of the implant in the present study was decided to be in the metaphysis close to the joint. This part of the bone is highly interesting for orthopedic questions regarding joint replacement and has been used in rats only by few authors so far [4, 5, 12].

In the present study we were able to create osteomyelitis in all infected tibiae with low amounts of bacteria (10^2^ and 10^3^ CFU). Numerous animal models creating osteomyelitis in rats, mice, and rabbits are using rather high bacterial concentrations (>10^3^ CFU) in order to create high numbers of infected animals [4, 9, 21–26]. The advantage in these studies is that nearly all animals develop infection and therefore evolve rather similarly, making comparison between groups and statistical analysis possible. However, the big disadvantage is that high bacterial loads do not mimic clinical constellations, neither of primary nor revision surgery. Even after eradication of PII and reimplantation of an orthopedic implant a rather sterile environment and only small amounts of residual, undetectable bacteria have to be assumed. Therefore, it is difficult to consider animal models creating infections by using inoculation loads far beyond 10^3^ CFU as studies mimicking clinically relevant situations. On the other hand, as shown in the present study, if low amounts of bacteria are used according to our approach local infection can reliably be induced. In this context, there are mainly three possible reasons why our model achieved those high rates of osteomyelitis: 1) The “catching effect” of the screw implant on the bacterial solution prevented loss of bacteria into the surrounding tissue. 2) The bacterial strain: *S. aureus (ATCC25923)* seems to be well suited for infect models as it was previously reported with high rates of osteomyelitis in an animal model [4]. Many other authors used different strains of *S. aureus* with considerably lower rates of infections [9, 10, 12, 16, 27]. 3) In the preparation of bacterial inocula we strictly avoided freezing of aliquots the day before implantation. This allows bacteria to be in maximum logarithmic growth at time of surgery and prevents from injecting bacteria not being active enough to resist the host’s antibacterial defense mechanisms.

Another finding in our study was the fact that we were able to establish constant bacterial presence on the implant and the adjacent bone and could demonstrate growth to common steady state value irrespective of the initial inocula, which differed by one order of magnitude (Table 6). These results are supported by Haenle and can be explained by a consistent environmental situation in all cases, meaning comparable nutritional supply and amount of host defense mechanisms [4]. The results indicate the achievement of a stationary phase of bacterial population during implant infection without systemic illness – comparable to the so-called “low-grade” infection. Apparently, this ultimate population size appears to be earlier reached on the implant surface than the peri-implant bone structures. Contradicting to these findings Lucke et al. reported an inoculation-dose depending amount of bacteria on the day of sacrifice [5].

The microbiological and histological evidence of infection in our study was only partially confirmed by radiological investigations. It is well known that conventional radiography is variable and unspecific in detecting osteomyelitis in early stages and therefore inferior to microbiological and histological evaluation of implant-associated infections, especially in cases where low bacterial concentrations are used to create infection [4, 28]. On the other hand, if high bacterial loads are used X-ray examinations can be quiet sensitive in depicting infection [6, 12]. A new approach in visualizing infection-induced osteolysis with higher sensitivity than conventional radiography can be the use of microCT imaging [9].

A second focus of the present work was on the use of low-dose Ag as an antibacterial additive on HA-coatings. Low-dose Ag was used to possibly provide sufficient osseointegration since the bactericidal activity of Ag has been stated to be present at concentrations as low as 35 ppb [29]. Ag^+^ concentrations in the powder of the HA-Ag coatings of the present study were 45 ppb and therefore rather low. In this context, a previous study evaluating similar coatings showed high bactericidal activity of HA-Ag samples in-vitro at short time [14]. On the other hand, still contradicting data exist regarding the reproducibility of antibacterial effects of Ag in-vitro and in-vivo showing that effects in-vitro do not necessarily have to be evident in-vivo [24]. However, such Ag coatings have rarely been applied to the surface of implants in the region of direct bone contact because of potential toxicity [13, 30]. To resolve this problem, we used HA as a support material for Ag^+^, because it offers good biocompatibility and osteoconductivity. Nevertheless, our study failed to show bactericidal effects of HA-Ag compared to untreated HA implants (Tables 5 and 6). However, all HA and HA-Ag implants of the sterile site showed excellent osseointegration (Table 7, Fig. 8). The sterilely inserted HA- and HA-Ag-screws were encapsulated by dense bone after 6 weeks, similar to what has been described previously around titanium implants [31]. Results from conventional X-ray examinations revealed that the same osseointegration as on HA-screws took place around HA-Ag-screws (Table 7). This finding was confirmed semiquantitatively by measurement of resistance-to-removal. Histological examination revealed no cellular inflammation or foreign-body granuloma around silver-coated implants of the control site, trabeculae and bone cells were normal (Fig. 8).

We acknowledge limitations to our study. Firstly, we used only one type of bacteria in this study. Antimicrobial-coated implants should provide activity against not only the most common causative bacteria (i.e., *S. aureus*) but also other potential pathogens. However, it is impractical to examine all pathogens. Secondly, our study is short-term and it is difficult to speculate whether the localized infection will show high-grade infection with sepsis in the long term. The length of the study was according to most authors studying implant-associated infection in animal models and therefore comparable to other studies. Thirdly, our biomechanical testing was semiquantitative by evaluating resistance-to-removal of the screws. This method was chosen due to the small sample sizes of the groups where quantitative measurements are difficult to state. Additionally, even if quantitative methods are used (e.g., resonant frequency analysis) correlation between biomechanical testing and osseointegration is not always observed [32, 33]. However, due to the fact that the screw with its internal thread could be easily connected with a customized torsiometer quantitative measurements would be performable in future studies focused primarily on questions regarding osseointegration.

## Conclusion

The findings of the present study highlight three main features: 1) Inoculation of small amounts (10^2^ and 10^3^ CFU) of *S. aureus* around a screw-shaped implant in the metaphysis of rat tibiae yielded to local infection in all animals. These bacterial loads are closer to clinical settings compared to inoculation of high doses exceeding 10^3^ CFU; 2) Excellent osseointegration of all implants (HA and HA-Ag) was present at the uninfected site indicating good biocompatibility of the coatings; 3) No antibacterial effect of low-concentrated HA-Ag coatings was found; 4) Presence of infection diminishes osseointegration.

This animal model can be considered suitable for studies on the efficacy of prophylaxis, treatment, and pathogenesis of implant-related infections of bone and may serve for investigations analyzing the influence of various implant surface properties on the development of bone infection.

### Data availability

The datasets supporting the conclusions of this article are included within the article.

## Abbreviations

ABR: attached bone remnants
Ag: silver
Ag^+^: silver ions
CFU: colony-forming units
HA: hydroxyapatite coating
HA-Ag: hydroxyapatite-silver coating
PII: peri-implant infection
RTR: resistance-to-removal
S. aureus: staphylococcus aureus
